# Predictive Biomarkers of Dicycloplatin Resistance or Susceptibility in Prostate Cancer

**DOI:** 10.3389/fgene.2021.669605

**Published:** 2021-07-27

**Authors:** Minglu Liu, Xiaoyu Zhou, Jun Liu, Chelong Lu, Guoqing Zhang, Jing Zhang, Shunchang Jiao

**Affiliations:** ^1^Department of Medical Oncology, Chinese PLA General Hospital, Beijing, China; ^2^GenomiCare Biotechnology Co., Ltd., Shanghai, China

**Keywords:** prostate cancer, dicycloplatin, whole-exome sequencing, biomarker, anti-cancer (anticancer) drugs

## Abstract

**Background:**

Prostate cancer (PCa) is among the leading causes of cancer mortality. Dicycloplatin is a newer generation platinum-based drug that has less side effects than cisplatin and carboplatin. However, its effects in PCa is mixed due to lack of appropriate stratifying biomarkers. Aiming to search for such biomarkers, here, we analyze a group of PCa patients with different responses to dicycloplatin.

**Methods:**

We carried out whole-exome sequencing on cell-free DNA (cfDNA) and matched leukocyte DNA from 16 PCa patients before treatment with dicycloplatin. We then compared the clinical characteristics, somatic mutations, copy number variants (CNVs), and mutational signatures between the dicycloplatin-sensitive (nine patients) and dicycloplatin-resistant (seven patients) groups and tested the identified mutations, CNV, and their combinations as marker of dicycloplatin response.

**Results:**

The mutation frequency of seven genes (*SP8*, *HNRNPCL1*, *FRG1*, *RBM25*, *MUC16*, *ASTE1*, and *TMBIM4*) and CNV rate of four genes (*CTAGE4*, *GAGE2E*, *GAGE2C*, and *HORMAD1*) were higher in the resistant group than in the sensitive group, while the CNV rate in six genes (*CDSN*, *DPCR1*, *MUC22*, *TMSB4Y*, *VARS*, and *HISTCH2AC*) were lower in the resistant group than in the sensitive group. A combination of simultaneous mutation in two genes (*SP8*/*HNRNPCL1* or *SP8*/*FRG1*) and deletion of *GAGE2C* together were found capable to predict dicycloplatin resistance with 100% sensitivity and 100% specificity.

**Conclusion:**

We successfully used cfDNA to monitor mutational profiles of PCa and designed an effective composite marker to select patients for dicycloplatin treatment based on their mutational profile.

## Introduction

Prostate cancer (PCa) is one of the most common cancers in men worldwide and has the second highest mortality (Siegel et al., 2020). It was estimated that there would be about 190,000 new cases of PCa (21% of all are male cancers) and 30,000 deaths (10% of all are male cancer deaths) in the United States alone in 2020 (Siegel et al., 2020). Hormone therapy is an effective therapy that can improve the survival time and clinical benefits for early stage PCa. However, about 10–20% of patients will inevitably develop into drug resistance within 5 years during the course of treatment, leading to castration-resistant prostate cancer (Kirby et al., 2011). Platinum-based chemotherapies, such as cisplatin and carboplatin, are attracting more and more attention in the treatment of cancer (Apps et al., 2015). These drugs mainly function through induction of DNA cross-links, therefore inhibiting DNA synthesis, mitosis, and induce apoptosis (Lokich and Anderson, 1998; Ozols et al., 2003). However, the clinical benefits from these drug therapies are still low, they can prolong patients’ overall survival for only 3–6 months (Fortin et al., 2013). Moreover, their clinical application is limited by severe adverse effects, including ototoxicity, neurotoxicity, and myelosuppression (Rossi et al., 2012). Therefore, there is an urgent need to develop more effective drugs to PCa with lower side effects.

Dicycloplatin is a derivative of carboplatin in which a carboxylic acid ligand is bound to the carboplatin moiety through hydrogen bonds. Therefore, it has a more stable chemical structure and better aqueous solubility than carboplatin (Yang et al., 2010). Previous studies showed that dicycloplatin has a better anticancer activity and much lower toxicity than cisplatin and carboplatin in patients with non-small cell lung cancer (Wang and Yu, 2004). *In vivo* and *in vitro* studies showed that dicycloplatin can induce cell cycle arrest and apoptosis and inhibit cell proliferation through reactive oxygen species stress-mediated death receptor pathway and mitochondrial pathway (Yu et al., 2014). Furthermore, a phase II clinical trial in non-small cell lung cancer has demonstrated the safety and efficacy of dicycloplatin in combination with paclitaxel (Liu et al., 2014). However, a considerable number of patients did not benefit from dicycloplatin treatment with some unknown reasons. Effective and reliable prognostic factors, therefore, are desperately needed in order to target dicycloplatin to the subset of patients who would benefit most from the treatment.

Whole exome sequencing (WES) is a high-throughput sequencing technology that can explore the whole functional DNA sequence and genetics variations of each patient to uncover novel molecules that may be related to therapies. In this retrospective study, we carried out WES on cell-free DNA (cfDNA) from blood- and patient-matched leukocyte DNA in 16 PCa patients before they received dicycloplatin monotherapy. A comprehensive analysis was performed to search for the association between the clinical outcomes of dicycloplatin treatment and molecular characterization of the patients, such as somatic mutations, copy number variants, and mutational signatures.

## Materials and Methods

### Patients, Clinical Evaluation, and Sample Collection

Sixteen PCa patients were retrospectively enrolled from Chinese PLA General Hospital with the following criteria: (Siegel et al., 2020) Eastern Cooperative Oncology Group performance status of 0–2; (Kirby et al., 2011) patients with distant metastasis who have received surgical castration or medical castration (serum testosterone ≤ 50 ng/dl or 1.7 nmol/ml); (Apps et al., 2015) at least 4 weeks after antiandrogenic therapy; and (Lokich and Anderson, 1998) the value of prostate specific antigen (PSA) was more than 2 ng/ml, and sustained increase more than 50% one week. The patients were on dicycloplatin treatment during 2016–2019. The study protocol was approved by the Ethics Committee of Chinese PLA General Hospital (approval number S2017-032-02). All patients provided a written informed consent.

The pathological diagnosis was performed by experienced pathologists of the hospital. Tumor response to the treatment was evaluated based on the patients’ radiological images [computed tomography (CT) and magnetic resonance imaging (MRI)] according to the Response Evaluation Criteria in Solid Tumors, version 1.1 (Tirkes et al., 2013).

### Whole-Exome Sequencing

Ten milliliter of blood specimens was collected before dicycloplatin treatment and used to prepare cfDNA by MagMAX(Cell-Free DNA Isolation Kit (Applied Biosystems(A29319) and leukocyte DNA by Maxwell^®^ RSC Blood DNA Kit (Promega AS1400). cfDNA for WES needed to meet the following conditions: there is an obvious peak in the region of 100–300 bp, and the regional molarity of the peak was >0.7 of all contents between 100 bp and 42 kb (indicating that there is no contamination from large genomic DNA). The purified DNA was sonicated using a Covaris L220 sonicator and hybridized to the probes in SureSelect Human All Exon V5 kit (cat. # 5190-6209 EN, Agilent Technologies, Sta. Clara, CA, United States) to capture exonic DNA, then prepared to libraries using the SureSelectXT Low Input Target Enrichment and Library Preparation system (cat. # G9703-90000, Agilent Technologies). Paired end reads of 150 × 150 bp were generated from the libraries using an Illumina NovaSeq-6000 sequencer (Illumina, San Diego, CA, United States). Image analysis and base calling were done using the onboard RTA3 software (Illumina). After removing adapters and low-quality reads, the reads were aligned to National Center for Biotechnology Information (NCBI) human genome reference assembly hg19 using the Burrows–Wheeler Aligner alignment algorithm and further processed using the Genome Analysis Toolkit (GATK, version 3.5), including the GATK Realigner Target Creator to identify regions that needed to be realigned. Somatic mutations, including single-nucleotide variants (SNVs), indel, and copy number variation (CNV) were determined by a comparison between the aligned sequences from cfDNA and patient-matched leukocyte DNA using the MuTect/ANNOVAR/dbNSFP31, VarscanIndel, and CNVnator software, respectively, as previously reported (Zang et al., 2019). The mutational signature classification was based on COSMIC Mutational Signature (version 2—March 2015), which was generated from studies performed by others (Maitra et al., 2013; Alexandrov et al., 2015; Nik-Zainal et al., 2016). Tumor mutation burden (TMB) was defined as the total number of somatic non-synonymous mutations in each sample according to a previous method for WES data (Chalmers et al., 2017). All autosomal microsatellite tracts containing 1--5 bp repeating subunits in length and comprising five or more repeats in GRCh37/hg19 were identified using MISA^1^ and used to calculate microsatellite instability score (MSI). MSI score was calculated by the number of unstable microsatellite sites/total valid sites.

The WES data of each patients were submitted in NCBI with submission number SUB9593847 and accession number PRJNA727718.

### Statistical Analysis

All statistical analyses were performed using R^2^ or SPSS 25 for Windows (SPSS Inc., Chicago, IL, United States). Differences in the distribution of somatic mutations, mutational signatures, and clinical characteristics between patient subgroups were evaluated by the Fisher’ exact test and Mann–Whitney *U* test for categorical and continuous parameters, respectively, and events of *p* < 0.05 were considered statistically significant.

## Results

### Clinicopathological Characteristics of the Patients

**FIGURE 1:**
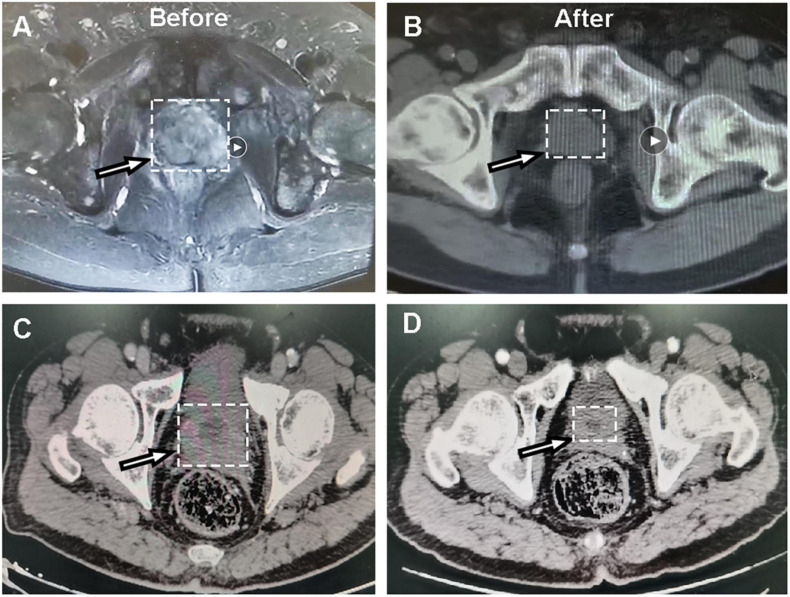
Representative CT images of dicycloplatin-sensitive patients. **(A,B)** CT images of one patient with complete response taken before **(A)** and after **(B)** dicycloplatin treatment. **(C,D)** CT images of another patient with partial response taken before **(C)** and after **(D)** dicycloplatin treatment. White arrow represents the tumor localization.

In total, 16 patients treated with dicycloplatin were enrolled, and their baseline characteristics are shown in Table 1. Nine of the patients were sensitive to the treatment, including six with partial response (PR) and three with complete response (CR) evaluated by CT and MRI scans. The remaining seven patients were resistant to the treatment, including six with progressive disease (PD) and one with stable disease (SD). The overall tumor response rate was 56.3%. The representative diagnostic images are shown in Figure 1. The PSA and free PSA (fPSA) levels of each patients during treatment course were analyzed; both PSA and fPSA were decreased in patients with PR and CR, while they were increased in patients with PD and SD (Supplementary Figure 1). Patients who received endocrine therapy before dicycloplatin treatment were more likely resistant to dicycloplatin (Table 1, Fisher’s exact test, *p* = 0.003). Other factors, such as age, smoking and drinking history, tumor stage, Gleason score, pre-dicycloplatin treatment history of surgery, chemotherapy, and radiotherapy, all showed no significant difference between the dicycloplatin-sensitive and dicycloplatin-resistant groups.

**TABLE 1:** Comparison of baseline characteristics between dicycloplatin-sensitive and dicycloplatin-resistant patients.

**Baseline characteristic**	**Sensitive (*n* = 9)**	**Resistant (*n* = 7)**	***p* value**	**All patients (*n* = 16)**
Age, median (range), years	66 (61–79)	63 (51–72)	0.210	66 (51–79)
**Smoking history, no. (%)**			0.633	
Yes	4 (44.4)	2 (28.6)		6 (37.5)
No	5 (55.5)	5 (71.4)		10 (62.5)
**Drinking history, no. (%)**			0.596	
Yes	2 (44.4)	3 (42.9)		6 (37.5)
No	7 (55.5)	4 (57.1)		10 (62.5)
**Tumor stage at diagnosis, no. (%)**			1	
II	1 (11.1)	0 (0.0)		1 (6.3)
IV	8 (88.8)	7 (100.0)		15 (93.8)
**Gleason sum at diagnosis, no. (%)**			1	
≤7	6 (66.6)	4 (57.1)		10 (62.5)
≥8	3 (33.3)	3 (42.9)		6 (37.5)
**Prior treatment for PCa, no. (%)**				
Surgery	2 (22.2)	1 (14.3)	1	3 (18.8)
Radiotherapy	1 (11.1)	2 (28.6)	0.4	3 (18.8)
Chemotherapy	3 (33.3)	5 (71.4)	0.157	10 (62.5)
Endocrine therapy	2 (22.2)	7 (100.0)	0.003	9 (56.3)
Pretreatment PSA level, median (range), ng/ml	87.6 (9.9–775.6)	79.3 (2.9–800)	0.837	86.0 (2.9–800)
Pretreatment-free PSA level, median (range), ng/ml	14.0 (0.5–48.6)	10.8 (0.9–29.8)	0.681	11.2 (0.5–48.6)

*PSA, prostate-specific antigen. *p* values are based on Fisher’ exact test and Mann–Whitney *U* test for categorical and continuous parameters, respectively.*

### The Mutational Landscape of the Patients

**FIGURE 2:**
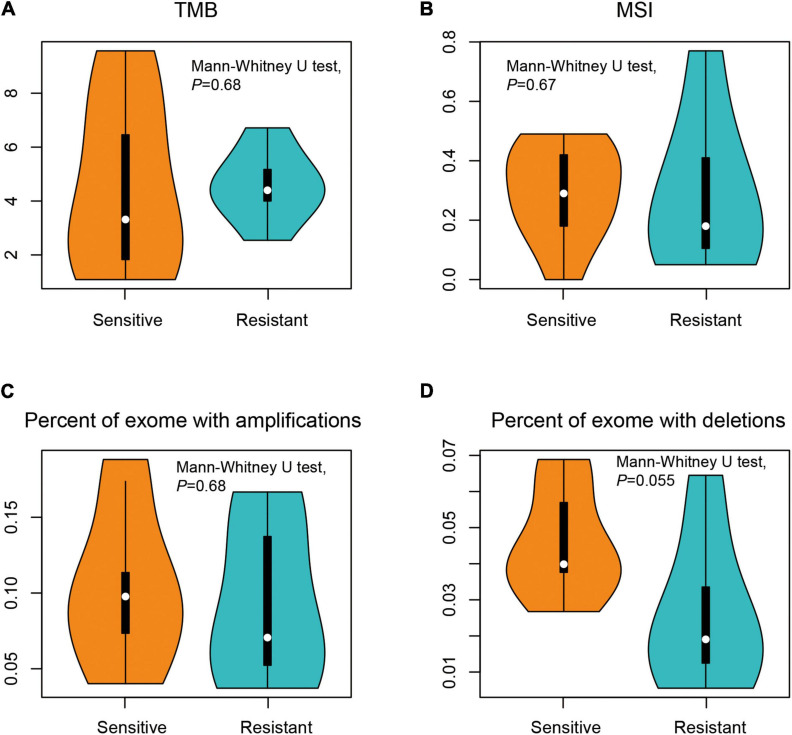
Comparison of the genomic profiles between dicycloplatin-sensitive and dicycloplatin-resistant groups. **(A)** Tumor mutational burden (TMB). **(B)** Microsatellite instability (MSI) score. **(C)** Percentage of exons with amplification (defined as copy number ≥ 2 × average ploidy). **(D)** Percentage of exons with deletion (defined as copy number ≤ 0.5 × average ploidy).

The whole-exome DNAs of the patients were captured and sequenced on an Illumina platform. The average sequencing depth was ×925 for cfDNA libraries and ×117 for leukocyte DNA libraries. The sequences from cfDNA were compared to matched leukocyte DNA to give somatic genetic changes including SNV, indel, and CNV (see section “Materials and Methods”). The median TMB was similar between treatment-sensitive group (3.3 mutations/Mb) and treatment-resistant group (4.4 mutations/Mb), with no statistical difference (Mann–Whitney *U* test, *p* = 0.680). There were also no differences in MSI score, the proportions of gene amplifications, and deletions between the two groups (Mann–Whitney *U* test, *p* > 0.05; Figure 2).

**FIGURE 3:**
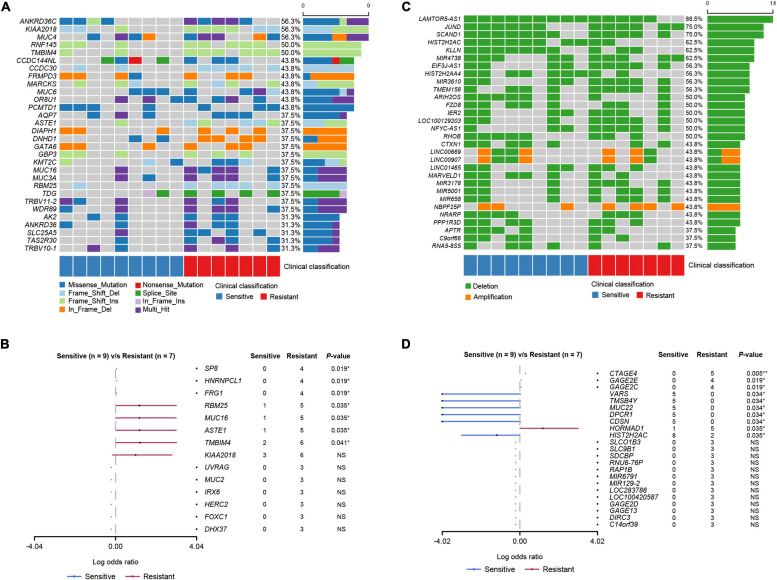
Mutational landscape of the PCa patients. **(A)** The top 30 genes with SNV and Indel mutations. The percentage and horizontal bar on the right of each row indicate the fraction of patients with mutations in the corresponding genes and the composition of the types of mutations as color coded below the plot. **(B)** Somatic differentially mutated genes between the dicycloplatin-sensitive and dicycloplatin-resistant groups. The dots and horizontal bars denote the hazard rate and 5–95% confidence interval (CI), respectively. **(C)** The top 30 genes with CNV. The percentage and horizontal bar on the right of each row indicate the fraction of patients with CNV in the corresponding genes and composition of amplification (orange) or deletion (green). **(D)** Genes with differential CNV between the dicycloplatin-sensitive and dicycloplatin-resistant groups. The patients are grouped by their response to dicycloplatin. Blue: sensitive; red: resistant. The dots and horizontal bars denote the hazard rate and 5–95% CI in **(B,D)**. **p < 0.01, *p < 0.05. NS, not significant.

The most common somatic mutated genes were *ANKRD36C* (56.3%), *KIAA2018* (56.3%), *MUC4* (56.3%), *TMBIM4* (50.0%), and *RNF145* (50.0%) among all 16 patients (Figure 3A). We identified seven genes whose mutation rates were significantly higher in the resistant group than in the sensitive group: *SP8*, *HNRNPCL1*, *FRG1*, *RBM25*, *MUC16*, *ASTE1*, and *TMBIM4* (Fisher’s exact test, *p* < 0.05; Figure 3B). The mutation rates of *SP8*, *HNRNPCL1*, and *FRG1* were 57.1% (4/7) in the sensitive group and 0% in the resistant group. The mutation rates of *ASTE1*, *MUC16*, and *RBM25* were 71.4% (5/7) in the sensitive group and 11.1% (1/9) in the resistant group.

All mutated genes were screened for a possible link to signaling pathways associated with platinum metabolism curated from the literature, such as cell cycle dependence, bicyclic platinum molecule activation, DNA damage repair, tumor cell apoptosis, drug transmembrane transport, platinum metabolizing drugs, DNA homeostasis disorders, and potential secondary drug resistance. The result showed that the mutation rates of these genes were similar in two groups, except for *MUC16* (Supplementary Figure 2).

### Copy Number Variations

To further explore events that are related to dicycloplatin treatment, we analyzed CNVs harbored by the patients. *LAMTOR5-AS1*, *JUND*, and *SCAND1* were the most common genes with CNV deletion, and the number of CNV deleted genes was greater than the number of CNV-amplified genes (Figure 3C). The variation rates of CNV of four genes (*CTAGE4*, *GAGE2E*, *GAGE2C*, and *HORMAD1*) were higher in the resistant group than in the sensitive group (Fisher’s exact test, *p* < 0.05). In contrast, the variation rates of CNV of six genes (*VARS, TMSB4Y*, *MUC22*, *DPCR1*, *CDSN*, and *HISTCH2AC*) were lower in the resistant group than in the sensitive group (Fisher’s exact test, *p* < 0.05; Figure 3D).

### Mutational Spectrum and Mutational Signatures

**FIGURE 4:**
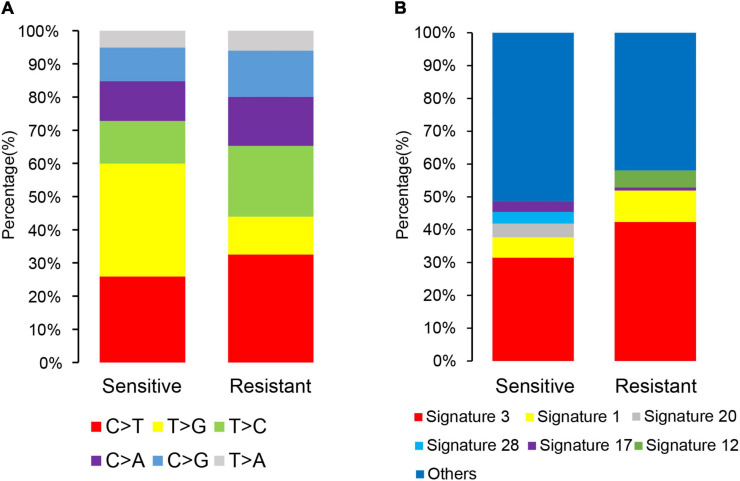
Single-nucleotide and composite mutational signatures in dicycloplatin-sensitive and dicycloplatin-resistant groups. **(A)** Stacked bar graph of the percentages of six single-nucleotide substitutions in the sensitive (left) and resistance (right) groups. **(B)** Stacked bar graph of the percentages of COSMIC trinucleotide mutational signatures in the sensitive (left) and resistance (right) groups. The types of substitutions are color coded.

A scan of the observed mutations in the patients showed that C>T was the most common substitution in the cfDNA samples (Figure 4A). T>G substitution was the most common of the six base substitutions in the sensitive group. Its proportion decreased in the resistant group, but there was no statistical difference (Mann–Whitney *U* test, *p* > 0.05). The COSMIC mutational signatures of the patients are presented in Figure 4B. Signatures 3 and 1 were dominant in all samples with median percentages at 40.1 and 8.1%, respectively. The median percentages of the other mutational signatures ranged from 0 to 2.1%. Signature 12 had a higher proportion in the resistant group than in the sensitive group (Mann–Whitney *U* test, *p* = 0.018), while the other signatures did not show statistical differences between these two groups (Mann–Whitney *U* test, *p* > 0.05).

### Performance of Gene Classifiers in Predicting Resistance or Sensitivity to Dicycloplatin

There were differences in the frequency of multiple gene mutations and CNV variants between the resistant group and the sensitive group. The mutation frequency of seven genes (*SP8*, *HNRNPCL1*, *FRG1*, *RBM25*, *MUC16*, *ASTE1*, and *TMBIM4*) and the CNV variation frequency of four genes (*CTAGE4*, *GAGE2E*, *GAGE2C*, and *HORMAD1*) were higher in the resistant group than in the sensitive group. In order to search for markers of dicycloplatin treatment effects, we first calculated the sensitivity and specificity of these genes individually in assessing dicycloplatin resistance (Table 2, Nos. 1–11). Individually, none of these genes can satisfy a high sensitivity and high specificity at the same time. The sensitivity of three mutated genes (*SP8*, *HNRNPCL1*, and *FRG1*) and two CNV genes (*GAGE2E* and *GAGE2C*) was 57.1%, and their specificity was 100.0%. The sensitivity and specificity of three other mutated genes (*RBM25*, *MUC16*, and *ASTE1*) were 71.4 and 88.9%, respectively. Of the 11 genes tested, *TMBIM4* had the highest sensitivity (85.7%), but its specificity was the lowest (77.8%). The sensitivity of the remaining two copy number variated genes (*CTAGE4* and *HORMAD1*) was 71.4%, and their specificities were 100 and 88.9%, respectively.

**TABLE 2:** The sensitivity and specificity of single gene in assessing dicycloplatin resistance (Nos. 1–11) and dicycloplatin susceptibility (Nos. 12–17).

**No.**	**Genes**	**TP**	**FN**	**FP**	**TN**	**Sensitivity**	**Specificity**	***p* value**
1	*SP8*	4	3	0	9	57.1%	100.0%	0.019
2	*HNRNPCL1*	4	3	0	9	57.1%	100.0%	0.019
3	*FRG1*	4	3	0	9	57.1%	100.0%	0.019
4	*RBM25*	5	2	1	8	71.4%	88.9%	0.035
5	*MUC16*	5	2	1	8	71.4%	88.9%	0.035
6	*ASTE1*	5	2	1	8	71.4%	88.9%	0.035
7	*TMBIM4*	6	1	2	7	85.7%	77.8%	0.041
8	*CTAGE4*	5	2	0	9	71.4%	100.0%	0.005
9	*GAGE2E*	4	3	0	9	57.1%	100.0%	0.019
10	*GAGE2C*	4	3	0	9	57.1%	100.0%	0.019
11	*HORMAD1*	5	2	1	8	71.4%	88.9%	0.035
12	*VARS*	5	4	0	7	55.6%	100.0%	0.034
13	*TMSB4Y*	5	4	0	7	55.6%	100.0%	0.034
14	*MUC22*	5	4	0	7	55.6%	100.0%	0.034
15	*DPCR1*	5	4	0	7	55.6%	100.0%	0.034
16	*CDSN*	5	4	0	7	55.6%	100.0%	0.034
17	*HIST2H2AC*	8	0	2	5	100.0%	71.4%	0.035

*TP, true positive; FN, false negative; FP, false positive; and TN, true negative. The sensitivity in percentage is derived from the equation [TP/(TP + FN)]. The specificity in percentage is derived from the equation [TN/(TN + FP)]. *p* values are based on Fisher’ exact test between the dicycloplatin-sensitive (nine patients) and dicycloplatin-resistant (seven patients) groups.*

The sensitivity and specificity of the CNV of six genes (*VARS*, *TMSB4Y*, *MUC22*, *DPCR1*, *CDSN*, and *HISTCH2AC*) in assessing dicycloplatin susceptibility were also calculated and shown on Table 2 (Nos. 12–17). The sensitivity of five copy number variated genes (*VARS*, *TMSB4Y*, *MUC22*, *DPCR1*, and *CDSN*) was 55.6%, and their specificity was 100%. The sensitivity and specificity of *HISTCH2AC* were 100 and 71.4%, respectively.

We next calculated sensitivity and specificity of genes in different combinations to assess dicycloplatin resistance and susceptibility. We limited the number of genes in the combination within 3. Among all the tested combinations, one configuration stood out. It consisted of mutations of two genes (either *SP8*/*HNRNPCL1* or *SP8*/*FRG1*) and CNV loss of *GAGE2C* and successfully detected resistant cases with 100% sensitivity and 100% specificity (Table 3, Nos. 1–14). Another configuration of CNV loss of *DPCR1* and *TMSB4Y* together could detect sensitive cases with 88.9% sensitivity and 100% specificity (Table 3, Nos. 15–18).

**TABLE 3:** Classifiers of several genes in combination to assess dicycloplatin resistance (Nos. 1–14) and dicycloplatin susceptibility (Nos. 15–18).

**No.**	**Genes**	**TP**	**FN**	**FP**	**TN**	**Sensitivity**	**Specificity**	***P* value**
1	*SP8/HNRNPCL1*	6	1	0	9	85.7%	100.0%	0.001
2	*SP8/FRG1*	6	1	0	9	85.7%	100.0%	0.001
3	*FRG1/HNRNPCL1*	6	1	0	9	85.7%	100.0%	0.001
4	*SP8/CTAGE4*	6	1	0	9	85.7%	100.0%	0.001
5	*HNRNPCL1/CTAGE4*	6	1	0	9	85.7%	100.0%	0.001
6	*SP8/GAGE2C*	6	1	0	9	85.7%	100.0%	0.001
7	*HNRNPCL1/ASTE1*	7	0	1	8	100.0%	88.9%	0.001
8	*FRG1/ASTE1*	7	0	1	8	100.0%	88.9%	0.001
9	*ASTE1/CTAGE4*	7	0	1	8	100.0%	88.9%	0.001
10	*ASTE1/HORMAD1*	7	0	1	8	100.0%	88.9%	0.001
11	*MUC16/GAGE2C*	7	0	1	8	100.0%	88.9%	0.001
12	*HNRNPCL1/GAGE2C*	7	0	1	8	100.0%	88.9%	0.001
13	*SP8/HNRNPCL1/GAGE2C*	7	0	0	9	100.0%	100.0%	0
14	*SP8/FRG1/GAGE2C*	7	0	0	9	100.0%	100.0%	0
15	*TMSB4Y/HIST2H2AC*	9	0	2	5	100.0%	71.4%	0.005
16	*CDSN/TMSB4Y*	7	2	0	7	77.8%	100.0%	0.003
17	*MUC22/TMSB4Y*	7	2	0	7	77.8%	100.0%	0.003
18	*DPCR1/TMSB4Y*	8	1	0	7	88.9%	100.0%	0.001

*TP, true positive; FN, false negative; FP, false positive; and TN, true negative. The sensitivity in percentage is derived from the equation [TP/(TP + FN)]. The specificity in percentage is derived from the equation [TN/(TN + FP)]. *p* values are based on Fisher’ exact test between the dicycloplatin-sensitive (nine patients) and dicycloplatin-resistant (seven patients) groups.*

## Discussion

In this study, we found several genes with mutation or CNV to predict the resistance or susceptibility to dicycloplatin in PCa and evaluated the patients’ response to a novel platinum drug. To our knowledge, this is the first predictive study in its category. Patients stratification by molecular subtyping would be helpful to improve the effectiveness of dicycloplatin in PCa.

Whole exome sequencing analysis in solid tumors is normally done with tissue samples. However, in the case of PCa, due to both the nature of the organ and surgical manipulation involved, tissue sample is often unavailable. Therefore, we extracted cfDNA from the blood of the PCa patients and used that in WES analysis instead of tumor tissue. Similar application of WES using cfDNA has been reported before and detected SNV, CNV, mutational signatures, TMB, and other genomic parameters in a reasonable accuracy, as verified by a positive correlation between the analysis results from cfDNA and tumor tissue samples (Bos et al., 2020).

In this study, all the patients were classified as stage IV, except one patient who was classified as stage II. This implies that the samples used in the current study were mostly from advanced tumors with high circulating tumor DNA (ctDNA) fractions. Therefore, the sensitivity of identified prediction factors in WES is expected to match to that from tissue samples. Our result indicates that cfDNA is a promising surrogate of tumor tissue because cfDNA can be obtained through a minimal-invasive procedure (blood drawing) and at the same time has the advantage to overcome tumor heterogeneity (Gonzalez-Billalabeitia et al., 2019). We expect that the surrogate strategy may even have expanded clinical uses beyond screening biomarkers for dicycloplatin response.

Among the potential predictive genes of dicycloplatin response that we identified, *MUC16* has been extensively studied in several tumors. *MUC16* is known to promote the progression and metastasis of a variety of malignant tumors, and the abnormal expression of *MUC16* can lead to drug resistance to cytotoxic drugs and inhibition of apoptosis (Das et al., 2015). This is consistent with our findings that the mutation frequency of *MUC16* was higher in the dicycloplatin-resistant group. Most of the remaining genes that we identified have been found involved in tumorigenesis and disease progression. *SP8*, which encodes specificity protein 1/Klf-like zinc-finger transcription factor, inhibits KARS-mediated transformation and is also a tumor suppressor by itself (Fernandez-Zapico et al., 2011). The expression of SP8 was decreased in primary gastric cancer compared with normal gastric mucosa in a recent study (Chang et al., 2009). FRG1 expression was decreased in PCa tissues and that affected the migration and invasion of cancer cells (Tiwari et al., 2019). Deleterious mutations of FRG1, which had been identified in calcified pleura fibrous tumor and follicular thyroid cancer, were suggested to contribute to tumorigenesis (Erinjeri et al., 2018; Mehrad et al., 2018). Splicing regulator *RBM25* was identified as a tumor suppressor in acute myeloid leukemia, and the low level of RBM25 was associated with high MYC activity and poor prognosis of patients (Ge et al., 2019). In PCa, p53 regulates EMT by activating *RBM25*, thus promoting tumor progression and metastasis (Yang et al., 2019). However, the roles of the mentioned genes above in platinum metabolism and drug resistance remain unclear and need further investigation.

**FIGURE 5:**
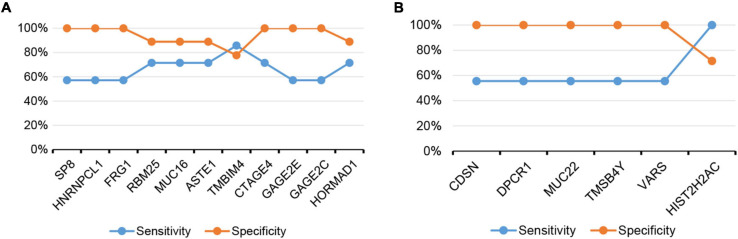
The sensitivity and specificity of assessing dicycloplatin response using the mutation or CNV status of single gene. **(A)** Dicycloplatin resistance and **(B)** dicycloplatin susceptibility.

The sensitivity and specificity to predict dicycloplatin response by the individual actionable genes did not reach 100% (Figure 5). From a clinical point of view, when the specificity of a screen is <100%, the patients who are false-positively identified can be tested by complementary methods and further cleared with their drug response status. However, if the sensitivity of a screen is <100%, the patients who are false-negatively identified will be missed in the screen. For oncologists and patients, the cost of suboptimal specificity is lower than the cost of suboptimal sensitivity. Therefore, it is preferable to evaluate patients’ drug response with a high-sensitivity screen. One way to increase the sensitivity is through the combination of multiple marker genes. We test several configurations and found the combinations of either *SP8/HNRNPCL1/GAGE2C* or *SP8/FRG1/GAGE2C* reached 100% in both sensitivity and specificity (Table 3). Although the sample size of this study is small, the putative drug response marker genes identified should provide a preliminary but critical assessment of the clinical value of dicycloplatin in PCa.

COSMIC mutational signature 25 had the higher proportion in the sensitive group than in the resistant group (Supplementary Figure 3). It was found highly represented in Hodgkin’s lymphoma, although its etiology remains unknown and needs further study.

In addition to the molecular markers, we also found that patients who had prior endocrine therapy were more likely to develop resistance to dicycloplatin (Table 1). It is possible that endocrine therapy and dicycloplatin resistance are mechanistically linked, probably due to clonal selection. However, another possibility is that the endocrine-treated patients were already in a more advanced stage when they received dicycloplatin, therefore had a worse clinical performance overall.

In conclusion, our study identifies that mutation or CNV in several genes are putatively predictive to dicycloplatin response in PCa. However, further tests in cell and animal models are necessary to search for and verify the possible action mechanism of these genes in platin drug resistance. The prediction method that we postulated should be valuable to screen patients suitable for dicycloplatin treatment, therefore reducing the suffering of PCa patients who are predicted as not good responders of the therapy.

## Data Availability Statement

The datasets presented in this study can be found in online repositories. The names of the repository/repositories and accession number(s) can be found below: https://www.ncbi.nlm.nih.gov/bioproject/PRJNA727718.

## Ethics Statement

Written informed consent was obtained from the individual(s) for the publication of any potentially identifiable images or data included in this article.

## Author Contributions

ML, CL, and GZ carried out carried on the study design and contributed to sample preparation. ML, XZ, and JZ analyzed the clinical data and wrote manuscript. JL and SJ revised the manuscript and approved final manuscript. All authors contributed to the article and approved the submitted version.

## Conflict of Interest

XZ, JL, and CL were employed by company GenomiCare Biotechnology Co., Ltd., Shanghai, China. The remaining authors declare that the research was conducted in the absence of any commercial or financial relationships that could be construed as a potential conflict of interest.

## Publisher’s Note

All claims expressed in this article are solely those of the authors and do not necessarily represent those of their affiliated organizations, or those of the publisher, the editors and the reviewers. Any product that may be evaluated in this article, or claim that may be made by its manufacturer, is not guaranteed or endorsed by the publisher.

## Footnotes

1
http://pgrc.ipk-gatersleben.de/misa/misa.html

2
https://cran.r-project.org

## Supplementary Material

Supplementary Figure 1 Dynamics of PSA and free PSA (fPSA) of patients during treatment course. PSA **(A)** and fPSA **(B)** levels were decreased in 6 PR patients and 3 CR patients, were increased in 6 PD patients and 1 SD patient.

Supplementary Figure 2 Mutational landscape of selected genes associated with tumor and platinum metabolism. **(A)** Oncoplot of the top 20 mutated gene in dicycloplatin-treated samples. The percentages and the bar plots on the right represent the fraction and the number of samples with variations in the corresponding gene. **(B)** Differentially somatic mutated genes between dicycloplatin-sensitive group and dicycloplatin-resistant group. The dots and horizontal bars denote the hazard rate and 5–95% CI. ^∗^*P*-value < 0.05. NS: not significant.

Supplementary Figure 3 Comparison of 30 cosmic signatures between dicycloplatin-sensitive and dicycloplatin-resistant patients.
