# Supplementary figures and images for: Predictive Biomarkers of Dicycloplatin Resistance or Susceptibility in Prostate Cancer
Source: Front Genet. 2021 Jul 27;12:669605. doi: 10.3389/fgene.2021.669605 (PMC8353331; doi:10.3389/fgene.2021.669605)

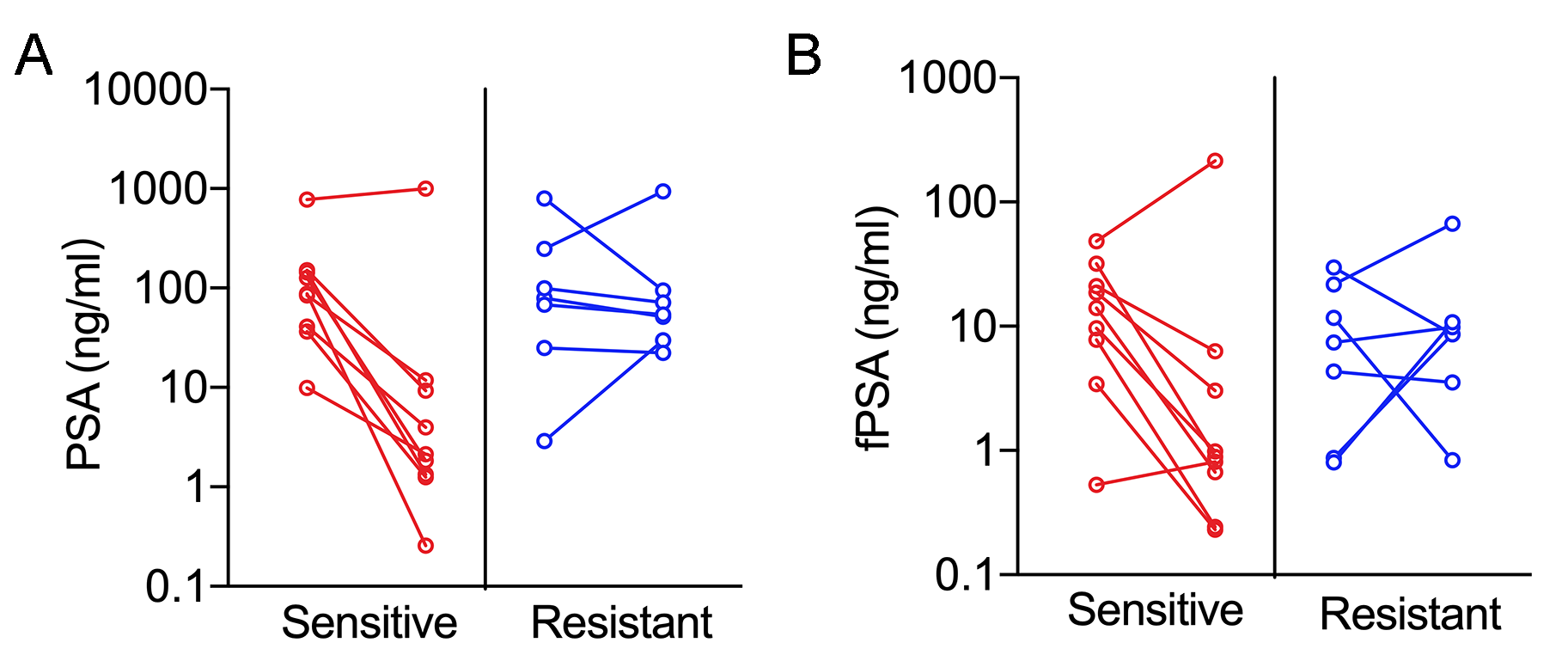

Supplement: Supplementary Figure 1 — Dynamics of PSA and free PSA (fPSA) of patients during treatment course. PSA (A) and fPSA (B) levels were decreased in 6 PR patients and 3 CR patients, were increased in 6 PD patients and 1 SD patient. [file Image_1.tif]

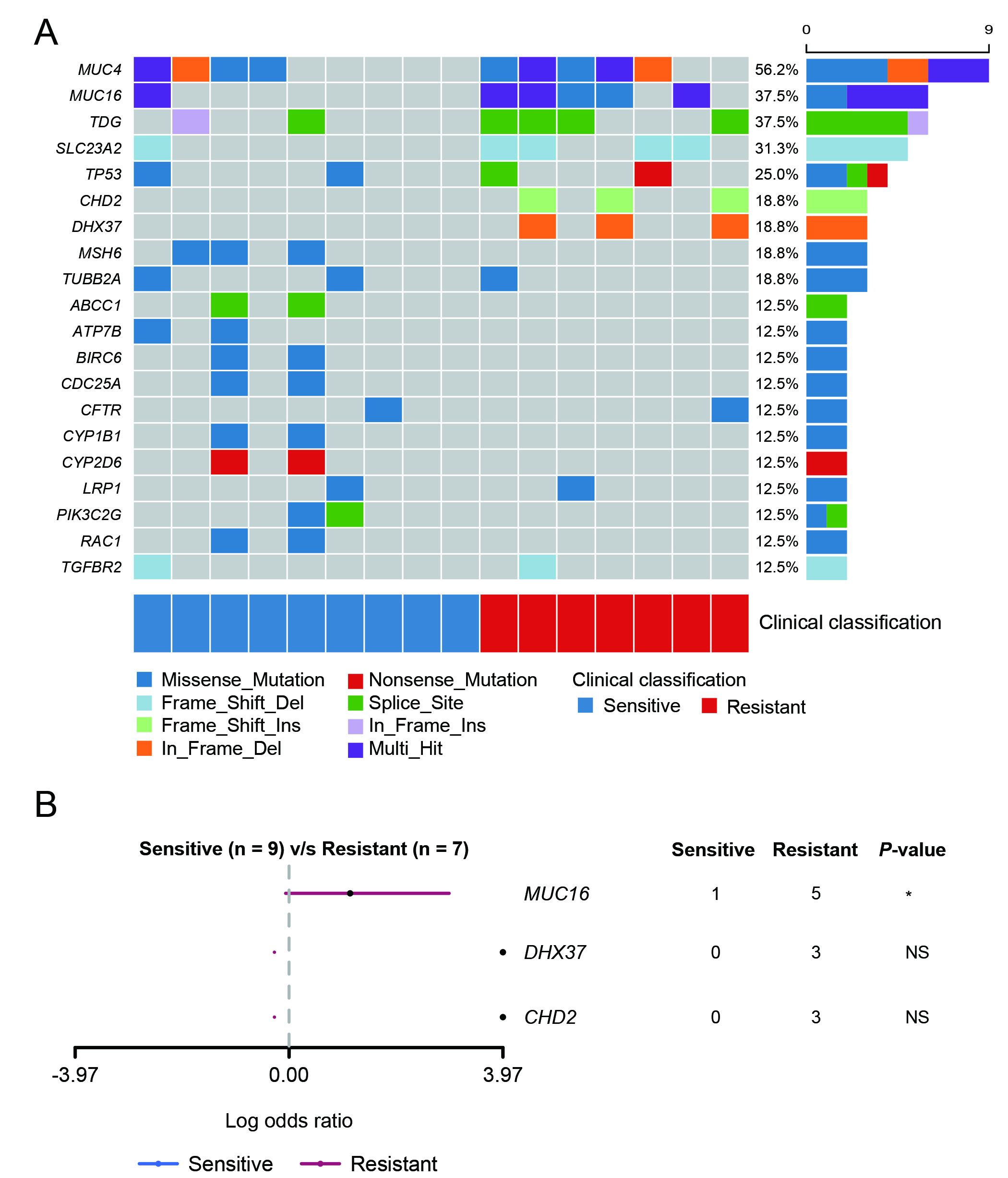

Supplement: Supplementary Figure 2 — Mutational landscape of selected genes associated with tumor and platinum metabolism. (A) Oncoplot of the top 20 mutated gene in dicycloplatin-treated samples. The percentages and the bar plots on the right represent the fraction and the number of samples with variations in the corresponding gene. (B) Differentially somatic mutated genes between dicycloplatin-sensitive group and dicycloplatin-resistant group. The dots and horizontal bars denote the hazard rate and 5–95% CI. ∗P-value < 0.05. NS: not significant. [file Image_2.tif]

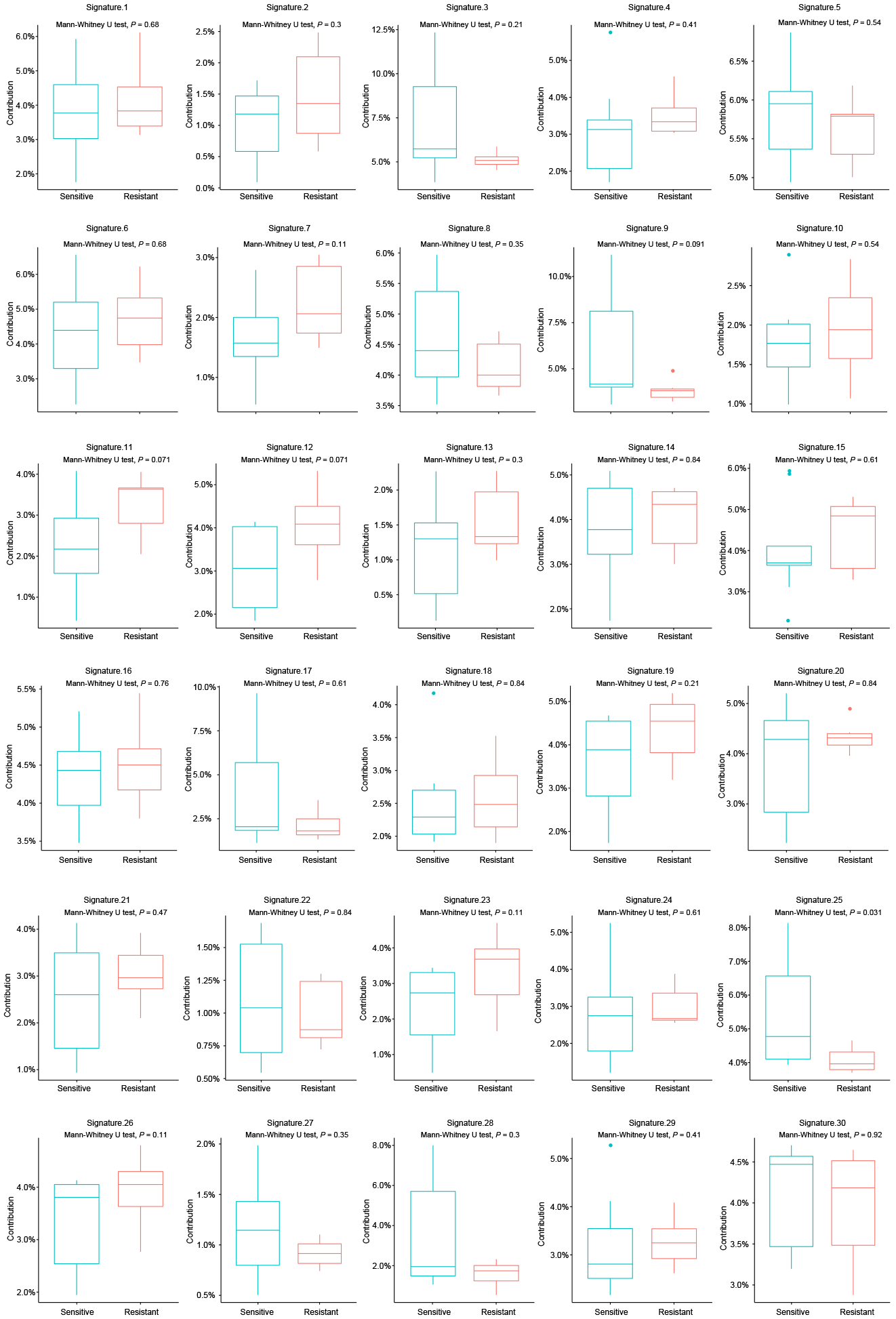

Supplement: Supplementary Figure 3 — Comparison of 30 cosmic signatures between dicycloplatin-sensitive and dicycloplatin-resistant patients. [file Image_3.tif]
